# Crystallographic Analysis of the Storage Protein Tub – a Tungstate Binding Protein from *Eubacterium limosum*

**DOI:** 10.1063/4.0001156

**Published:** 2025-10-27

**Authors:** Dayong Zhou, John P Rose, Nana Shao, Lirong Chen, Gerrit J. Schut, Farris L Poole, Michael W. W. Adams, B. C. Wang

**Affiliations:** University of Georgia, Athens, GA, USA

## Abstract

Enzymes containing tungsten are prevalent in the human gut microbiome and play an important role in maintaining overall gut health. A recent study of Eubacterium limosum revealed a new tungsten binding protein Tub that has nanomolar affinity for tungstate and contains a single TOBE domain found in molybdate storage proteins. A full paper about the protein structure has been published recently [Shao N, et. al, mBio, (2024)]. This poster describes the crystallographic aspects of structure solution and structural details.

To better understand Tug-tungstate binding and its structural similarity to molybdate storage proteins, high resolutioncrystal structures of Tug in its apo-form and three (5, 7 and 8) tungstate (WO4--) bound complexes were determined using an in-house Rigaku XtalLAB system. Similar to the molybdate binding proteins the 7 kD Tub protein monomer assembles as a hexamer consisting of a trimer composed of three Tub dimers.

The hexamer binds eight tungstate oxyanions located at inter-subunit interfaces. Two distinct tungstate binding sites were observed. Two, type-1 binding sites located at the top and bottom of the hexamer along the hexamer's non-crystallographic 3-fold axis and six, type-2 binding sites located on the dimer's 2-fold axis with each monomer containing a single site.

A comparison of the Tub type-1 and type-2 binding sites with those observed in other molybdenum binding proteins (ModG and MopII) showed that the two Tub type 1 binding sites were analogous to the type-1 sites found in ModG(PDB entry 1H9K) while the six Tub type-2 binding sites were similar to those found in MopII (PDB entry 1H9R).

The type-1 and type-2 sites were found to be linked by a hydrogen bond network (including a water molecule and the side chain of Asn 22) and are assessable via internal positively charged tunnels leading from the molecule's surface to the tungstate binding sites, whose surface entrance closes upon tungstate binding.

This work was funded by Bi-Cheng Wang's endowment fund for crystallographic studies, by John P. Rose's NIH (OD 021762) shared instrument grant for the UGA's Rigaku XtalLAB, and by Mike W.W. Adams' NIH (GM 136885) for the biochemical analysis.
